# Massively parallel sequencing of endometrial lavage specimens for the detection of cancer-associated mutations in atypical and non-atypical endometrial hyperplasia

**DOI:** 10.3389/fmed.2022.1090788

**Published:** 2022-12-22

**Authors:** Cindy Hsuan Weng, Kai-Yun Wu, Chin-Jung Wang, Huei-Jean Huang, Chia-Lung Tsai, Chiao-Yun Lin, Aileen Ro, Chyong-Huey Lai, An-Shine Chao, Ren-Chin Wu, Angel Chao

**Affiliations:** ^1^Department of Obstetrics and Gynecology, Linkou Chang Gung Memorial Hospital, Chang Gung University College of Medicine, Taoyuan, Taiwan; ^2^Gynecologic Cancer Research Center, Linkou Chang Gung Memorial Hospital, Taoyuan, Taiwan; ^3^Genomic Medicine Research Core Laboratory, Chang Gung Memorial Hospital, Taoyuan, Taiwan; ^4^Department of Obstetrics and Gynecology, New Taipei Municipal Tucheng Hospital, New Taipei City, Taiwan; ^5^Department of Pathology, Linkou Chang Gung Memorial Hospital, Chang Gung University College of Medicine, Taoyuan, Taiwan

**Keywords:** endometrial hyperplasia, atypia, endometrial lavage, office hysteroscopy, somatic mutations

## Abstract

**Background:**

Endometrial hyperplasia (EH), particularly with atypia, is considered an antecedent of endometrial adenocarcinoma. In this study, we aimed to apply massively parallel sequencing of endometrial lavage specimens for the detection of cancer-associated mutations in atypical (AEH) and non-atypical endometrial hyperplasia (NEH). The identified alterations were compared with those detected in tissue samples.

**Materials and methods:**

Endometrial lavage specimens and parallel biopsy samples (*n* = 11 for AEH and *n* = 9 for NEH) were obtained from 18 women (9 with AEH and 9 with NEH) who received an office hysteroscopy for suspected endometrial lesions. All samples were tested for somatic mutations in hotspot regions of 72 cancer-associated genes by massively parallel sequencing.

**Results:**

On analyzing sequencing data, the presence of at least one cancer-associated gene mutation was identified in 72.7 and 44.4% of endometrial lavage specimens obtained from women with AEH and NEH, respectively (*p* = 0.362, 95% confidence interval = 0.72-3.70). The concordance rates between mutations identified in endometrial lavage specimens and endometrial biopsies were 54.5 and 0% from women with AEH and NEH, respectively (*p* = 0.014). A patient with NEH harbored mutations in endometrial lavage with the same mutations found in the tissue specimen at low allele frequency below detection cutoff, raising the suspicion of missed focal atypia.

**Conclusion:**

Endometrial hyperplasia is characterized by a high burden of cancer-associated mutations, particularly in the presence of atypia. Our study, albeit performed with a relatively small number of samples, indicates that their detection by massively parallel sequencing of endometrial lavage is feasible. Our findings may allow tailoring of endometrial biopsies to the individual risk of AEH; additionally, they can pave the way toward less invasive surveillance protocols in patients with known EH.

## Introduction

Endometrial hyperplasia (EH) represents a spectrum of endometrial pathology defined by abnormal gland proliferation, architectural abnormalities, and an increased endometrial gland-to-stroma ratio (1). According to the World Health Organization (WHO) classification, EH can be categorized according to the presence or absence of atypia (2). EH, particularly with atypia, is considered a precursor of endometrial adenocarcinoma (1, 3). The estimated risk of malignant transformation for atypical hyperplasia (AEH) and non-atypical hyperplasia (NEH) over a 20-year period is estimated to be 28 and 5%, respectively (3, 4). Although there are significant differences between the WHO 1994 and endometrial intraepithelial neoplasia (EIN) classification systems of endometrial hyperplasia, a recent meta-analysis found that congruence with EIN criteria was high for complex AEH defined according to the WHO criteria (5). Additionally, AEH may coexist with endometrial cancer (EC) foci in up to 43% of cases (6).

In this scenario, a crucial aspect of clinical management is early detection and proper surveillance. Histological assessment *via* traditional hysteroscopy performed under general anesthesia is the gold standard for diagnosing EH. However, office-based hysteroscopy with endometrial biopsy has recently emerged as an effective tool for exploring the uterine cavity and obtaining pathological diagnosis (7–9). Unfortunately, no widely accepted criteria or established international guidelines currently exist to define the hysteroscopic appearance of AEH (10). It has been, therefore, suggested that the diagnostic capacity of hysteroscopy for the diagnosis of AEH is lower compared with EC (10).

Several fertility-preserving treatments (FPT)—including high-dose progestins (either with or without concomitant metformin) or levonorgestrel-releasing intrauterine systems—are currently available for women diagnosed with AEH (11–13). However, disease progression in patients who had undergone FPT may ultimately undermine therapies and compromise outcomes. Therefore, strict surveillance protocols consisting of repeated endometrial investigations are generally recommended (1). Unfortunately, multiple endometrial biopsies over time are associated with potential morbidity, including infectious and bleeding complications (14, 15). In recent years, the collection of uterine lavage samples performed during office-based hysteroscopy has emerged as a viable procedure for triaging diagnosis of endometrial lesions (16, 17). Furthermore, high-throughput genetic screening methods are particularly well suited for analyzing commonly mutated cancer driver genes in uterine lavage specimens (17, 18). We previously identified mutations in uterine lavage samples of endometrial cancer (19).

In this retrospective cohort study, we applied massively parallel sequencing of endometrial lavage samples collected during hysteroscopy to analyze mutations in women with AEH and NEH, and compared the detected mutations with those identified in parallel tissue samples. This information may allow tailoring of endometrial biopsy to the individual risk of AEH. This knowledge can also pave the way toward less invasive surveillance protocols in patients with known EH without resorting to serial tissue examinations.

## Materials and methods

### Design and ethics approval

This study was designed as a retrospective cohort study. All participants provided written informed consent to the investigation, and approval was granted by the Institutional Review Board at the Chang Gung Memorial Hospital (identifier: 202001329B0). The STROBE guidelines for cohort studies were followed for reporting.

### Participants

Women who attended for an office hysteroscopy between September 2020 and November 2021 were invited to participate. Eligibility criteria were abnormal uterine bleeding due to a suspected endometrial lesion or a known history of AEH managed using FPT. Patients with clinically manifest endometritis presenting with fever, pelvic pain, and increased vaginal discharge were excluded, as were those who were unwilling to participate (20). In an effort to minimize bias, we excluded all women for whom a final histological diagnosis was not achievable. Only women with a biopsy-proven diagnosis of AEH and NEH were deemed eligible. Age at onset, diagnosis achieved by pathological analysis of endometrial biopsies or hysterectomy specimens, and treatment data were collected from all participants.

### Collection of endometrial lavage samples

Office hysteroscopy was accomplished with the vaginoscopic technique without applying a vaginal speculum and/or a cervical clamp (7, 9). To distend and irrigate the uterine cavity, normal saline was instilled using an electronic infusion pump that kept an intrauterine pressure of 45 mmHg. Endometrial lavage samples (25 mL) were obtained with a 4-cm, continuous-flow rigid hysteroscopy system (Richard Wolf GmbH, Knittlingen, Germany) prior to endometrial biopsies. All specimens were centrifuged at 3,200 *g* for 20 min at 4°C within 2 h of collection. Cell pellets were washed with an erythrocyte lysis buffer and incubated at room temperature for 5 min. After removing the supernatant, cell pellets obtained from endometrial lavage were stored at −80°C until processing (17).

### Collection of endometrial biopsies and blood specimens

Endometrial biopsy specimens were collected during office hysteroscopy and processed to obtain formalin-fixed paraffin-embedded (FFPE) tissue blocks. Archived FFPE specimens were also retrieved for women with a pre-existing diagnosis of EH. Blood samples (10 mL) were drawn to ascertain whether variants identified in endometrial lavage samples or endometrial biopsies were somatic or germline.

### DNA extraction

Genomic DNA was extracted using the QIAamp DNA Mini Kit (Qiagen, Hilden, Germany). Tumor components were manually dissected from 10-μm-thick tissue sections. DNA concentrations and integrity were assessed using a Quant-iT dsDNA high-sensitivity assay kit (Invitrogen, Carlsbad, CA, USA) and a Fragment Analyzer (Advanced Analytical Technologies, Ankeny, IA, USA), respectively (21, 22).

### Massively parallel sequencing and mutation analysis

All coding exon sequences of the targeted genes were enriched using a polymerase chain reaction (PCR)-based strategy. Massively parallel sequencing (2 × 150 bp paired-end run) of collected specimens was carried out on a NextSeq 500 Sequencing System (Illumina, San Diego, CA, USA) using the AmpliSeq™ Cancer Hotspot Panel v2 (Illumina) that covered 72 cancer-associated genes (Supplementary Table 1). The uniformity of coverage for all samples was set at 95%. We filtered out all variants with a frequency of less than 1% in endometrial lavage samples (17) and 5% in tissue specimens (22). Raw reads were aligned to the hg19 reference genome. Annotation of all detected variants was carried out using the following packages: COSMIC (v. 81), East Asian population (ExAC_EAS), ClinVar database (23), gnomAD genome and exome databases, and 1000 Genomes Phase 3. Variants present in tissue samples but undetected in matched blood samples were considered as somatic mutations.

### Immunohistochemistry

Immunohistochemistry (IHC) was performed as previously described (24). In brief, sections were immunostained with an antibody against catenin beta-1 (CTNNB1; 1:200 dilution; Leica Biosystems, Vista, CA, USA) using a BOND Polymer Refine Detection system on an automated IHC stainer (Ventana, Tucson, AZ, USA).

### Statistical analysis

Patients with AEH and NEH were compared on categorical variables with the Fisher’s exact test. Analyses were performed using Statistical Program for Social Sciences (SPSS), version 26.0 (IBM, Armonk, NY, USA). All hypothesis testing was two-tailed, with statistical significance defined as a *p*-value < 0.05.

## Results

### Patient characteristics

Endometrial lavage specimens and parallel biopsy samples (*n* = 11 for AEH and *n* = 9 for NEH) were obtained from 18 women (9 with AEH and 9 with NEH). The clinical characteristics of the two study groups are summarized in Tables 1, 2, respectively. Two patients with AEH (EH11 and EH03) underwent definitive hysterectomy.

**TABLE 1:** Clinicopathologic characteristics of women with atypical endometrial hyperplasia.

Patient	Age (years)	BMI (kg/m^2^)	Histology		Treatment	Previous gynecologic history
			Current	Follow-up		
EH01	37	28.1	AEH	–	TCR + megestrol acetate 160 mg	NA
EH02	42	30.0	AEH	–	TCR + megestrol acetate 160 mg, metformin 1 g	NA
EH03	48	25.4	AEH	–	LAVH + BS	NEH
EH04 EH05	39	22.0	AEH	NEH	TCR + megestrol acetate 160 mg, metformin 1 g Mirena^®^	Cervical intraepithelial neoplasia
EH06 EH07	42	27.9	AEH	AEH	TCR + megestrol acetate 160 mg Mirena^®^	NA
EH09	33	44.2	AEH	–	TCR + megestrol acetate 160 mg, metformin 1 g Mirena^®^	NA
EH10	34	35.4	AEH	–	TCR	Polycystic ovarian syndrome
EH11	48	23.4	AEH	–	LAVH + BSO	Breast cancer
EH12	47	26.2	AEH	–	TCR	NA

BMI, body mass index; AEH, atypical endometrial hyperplasia; TCR, transcervical resection; NA, not applicable; NEH, non-atypical endometrial hyperplasia; LAVH, laparoscopic-assisted vaginal hysterectomy; BSO, bilateral salpingo-oophorectomy; BS, bilateral salpingectomy.

**TABLE 2:** Clinicopathologic characteristics of women with non-atypical endometrial hyperplasia.

Patient	Age (years)	BMI (kg/m^2^)	Histology		Treatment	Previous gynecologic history
			Current	Follow-up		
EH13	47	24.7	NEH	Secretory	TCR + megestrol acetate 80 mg, metformin 1 g	NA
EH14	51	19.8	NEH	Secretory	TCR	NA
EH15	51	28.0	NEH	–	Biopsy + medroxyprogesterone acetate 20 mg	NA
EH16	37	27.8	NEH	Proliferative	TCR + megestrol acetate 80 mg	Bilateral endometrioma, enucleation
EH17	47	20.8	NEH	NEH	Hysteroscopic removal + ablation	NA
EH18	33	21.3	NEH	–	TCR	TCR, myomectomy
EH19	41	20.1	NEH	–	TCR	TCR, myomectomy
EH20	48	22.2	NEH	–	Biopsy + Gynera^®^	NA
EH21	80	19.7	NEH	–	TCR	NA

BMI, body mass index; NEH, non-atypical endometrial hyperplasia; NA, not applicable; TCR, transcervical resection.

### Identification of mutations in endometrial lavage and tissue samples of women with AEH

**FIGURE 1:**
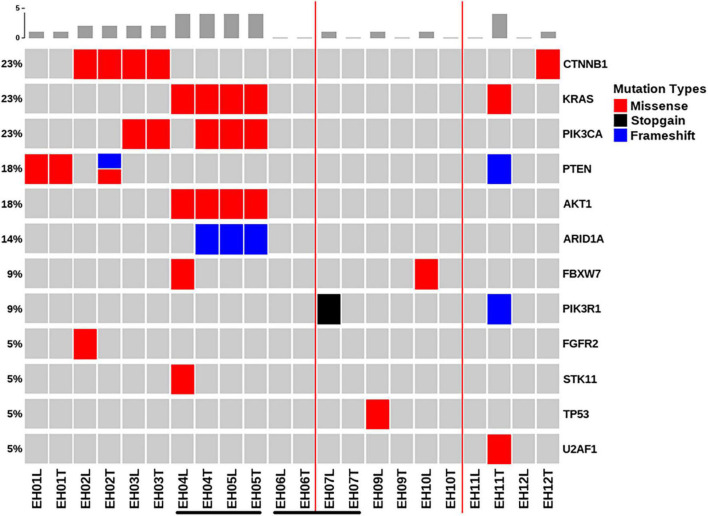
Distributions of gene mutations identified in endometrial lavage samples and tissue specimens obtained from women with atypical endometrial hyperplasia. Samples EH04 and EH05 were obtained from the same patient, as were samples EH06 and EH07. They are underlined with black lines. Red lines indicate the concordance category (mutations identified in endometrial lavage samples and tissue specimens; mutations in endometrial lavage samples only, and mutations in tissue specimens only). T, tissue specimen; L, endometrial lavage sample.

Figure 1 depicts all of the somatic mutations identified by massively parallel sequencing in women with AEH (*n* = 9). Follow-up samples were also obtained from two women (EH04, EH05; EH06, EH07) Supplementary Tables 2, 3. A total of 17 (allele frequency > 1%) and 22 (allele frequency > 5%) mutations were detected in endometrial lavage samples and tissue specimens, respectively. Specifically, 15 missense and 2 nonsense (1 premature stop codon and 1 frameshift) mutations were identified in endometrial lavage samples. In contrast, 16 missense and 6 nonsense (6 frameshift) mutations were detected in AEH tissue specimens.

Of the 11 endometrial lavage samples in nine patients with AEH, at least one mutation was identified in eight samples (detection rate: 72.7%). The detection rate of mutations was 63.6% in tissue specimens (Figure 1). The most commonly mutated genes were *CTNNB1*, *KRAS*, *PIK3CA*, *PTEN*, *AKT1*, and *ARID1A*. In sample-based analysis, the concordance of mutation patterns between endometrial lavage samples and biopsy specimens was 54.5%. Four patients with AEH (EH01, EH02, EH03, EH04, and EH05; with samples EH04 and EH05 being from the same patient) harbored somatic mutations in both endometrial lavage samples and tissue specimens. In one patient, no mutation was detected in both the endometrial lavage sample (EH06L) and the biopsy specimen (EH06T); however, a *PIK3R1* mutation was identified in an endometrial lavage sample (EH07L) collected during follow-up. Two patients harbored somatic mutations in endometrial lavage samples (EH09L and EH10L) but not in tissue specimens. Conversely, two patients had mutations in tissue specimens (EH11T and EH12T) but not in endometrial lavage samples.

### Identification of mutations in endometrial lavage and tissue samples of women with NEH

**FIGURE 2:**
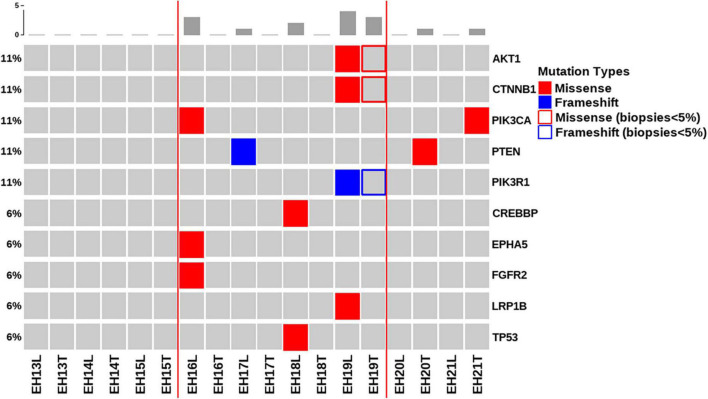
Distributions of gene mutations identified in endometrial lavage samples and tissue specimens obtained from women with non-atypical endometrial hyperplasia. Red lines indicate the concordance category (mutations identified in endometrial lavage samples and tissue specimens; mutations in endometrial lavage samples only, and mutations in tissue specimens only). The boxes indicate that the allele frequency of mutations in tissue samples was below the filtering threshold, while being present in lavage samples with a high allele frequency.

Figure 2 depicts all of the somatic mutations identified by massively parallel sequencing in women with NEH (*n* = 9), whereas detailed mutation data are listed in Supplementary Tables 2, 3. A total of 10 (allele frequency > 1%) and 2 (allele frequency > 5%) mutations were detected in endometrial lavage samples and tissue specimens, respectively. Specifically, eight missense and two nonsense (2 frameshift) mutations were identified in endometrial lavage samples. In contrast, two missense mutations were detected in tissue specimens.

**FIGURE 3:**
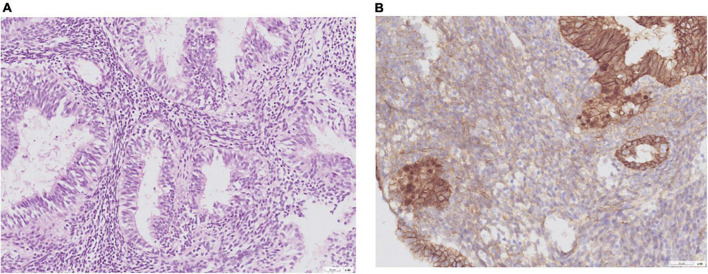
Representative histological images obtained from a woman with non-atypical endometrial hyperplasia (patient EH19). **(A)** Hematoxylin and eosin stain; **(B)** CTNNB1 immunostaining. A *CTNNB1* mutation was identified in the endometrial lavage sample but not in the corresponding tissue specimen. A review of pathological slides revealed suspected foci of atypia and prompted follow-up surveillance. This case illustrates the potential clinical impact of endometrial lavage sequencing.

Of the nine patients with NEH, four (detection rate: 44.4%) harbored somatic mutations in endometrial lavage samples (EH16L, EH17L, EH18L, and EH19L), including *TP53* missense mutations. Notably, the *AKT1* p.E17K and *CTNNB1* p.T41I mutations were identified in both endometrial lavage samples and tissue specimens (EH19); however, the allele frequency in tissue specimens was lower than the 5% threshold (Figure 2 and Supplementary Table 4). On IHC, the tissue specimen EH19T showed focal positive nuclear immunostaining for CTNNB1 (Figure 3). The remaining two patients harbored *PTEN* and *PIK3CA* mutations in tissue specimens (EH20T and EH21T, respectively).

On analyzing sequencing data, the presence of at least one cancer-associated gene mutation was identified in 72.7 and 44.4% of endometrial lavage specimens obtained from women with AEH and NEH, respectively (*p* = 0.362, 95% confidence interval = 0.72-3.70). The concordance rates between mutations identified in endometrial lavage specimens and endometrial biopsies were 54.5 and 0% from women with AEH and NEH, respectively (*p* = 0.014).

## Discussion

Clinical genomics studies using biofluids can contribute to improving the detection and risk stratification of precancerous conditions. Herein, we compared the mutational profiles of endometrial lavage samples and tissue specimens obtained from women with AEH and NEH. We found that EH was characterized by a relatively high burden of cancer-associated mutations, particularly in presence of atypia (detection rate in AEH: 72.7%). While a concordance between endometrial lavage and tissue mutational profiling was present in 54.5% of women with AEH, it was absent in NEH. In a case of NEH (EH19), mutations with high allele frequencies were identified in the endometrial lavage sample. The presence of the same mutations in the tissue sample, at a frequency lower than the filtering threshold, raised the suspicion of missed focal AEH. Although still limited in terms of sensitivity, we envisage that genetic profiling of endometrial lavage fluid obtained during office hysteroscopy may serve as a promising molecular approach for tailoring endometrial biopsy to the individual risk of AEH. In addition, our findings may help inform less invasive surveillance protocols in patients with EH who wish to receive FPT.

Multiple factors can contribute to the malignant transformation of EH into EC, including shared mutations in the *CTNNB1*, *PIK3CA*, *PTEN*, and *AKT1* genes (25, 26). While the mutation burden identified in AEH samples was higher than that detected in NEH, mutations in the *PIK3CA*, *PTEN* were present in both groups. These findings, different from previous studies reporting mutations identified in normal endometrium (27), clearly indicate that cancer-associated mutations can be found in EH even in the absence of atypia. This is in line with estimates derived from epidemiological studies showing that the risk of malignant transformation in NEH is low but not null (3, 4).

Our results revealed that, in certain cases, mutations not found or filtered out in EH biopsy specimens were identifiable using endometrial lavage samples; therefore, the combined analysis of the two matrices increased the number of mutations detected during screening. For example, the *AKT1* p.E17K and *CTNNB1* p.T41I mutations were filtered out due to low allele frequencies in a tissue specimen (EH19T) but were identified in the corresponding lavage sample (EH19L). Interestingly, IHC revealed that the EH19T specimen had focal positive nuclear immunostaining for CTNNB1; this finding validates the functional significance of the *CTNNB1* p.T41I mutation (28) detected in the lavage sample. Apart from the analytical aspects, endometrial lavage samples and tissue specimens may be discrepant for mutations because of intralesional heterogeneity. While larger studies are required before issuing clinical recommendations, serial genetic analysis of endometrial lavage samples may be more easily implemented during the course of FPT. Moreover, this approach can help monitor disease burden in conjunction with traditional endometrial biopsy.

The Cancer Genome Atlas (TCGA) has previously identified four distinct molecular signatures in endometrial cancer—termed POLE-mutated/ultramutated (POLEmt), microsatellite-instable/hypermutated (MSI), copy-number-high/p53-mutated (p53mut), and no specific molecular profile (NSMP) (25)—with prognostic implications. However, other risk factors—including lymphovascular space invasion—may have an impact on clinical outcomes (29). In an effort to further refine the clinical management of endometrial cancer, the European Society of Gynecological Oncology, the European Society for Radiotherapy and Oncology, and the European Society of Pathology have recently released joint guidelines to further integrate the TCGA signature with traditional prognostic factors (e.g., lymphovascular space invasion, histotype, and deep myometrial invasion) (29). In this scenario, we believe that our work has three main implications. First, our results indicate that the detection of mutations in endometrial lavage samples is feasible and allows triaging which patients should undergo endometrial biopsy. Second, mutation testing of endometrial lavage may promote a shift toward less invasive surveillance protocols for women with endometrial hyperplasia. Finally, the integration of molecular signatures may allow for the development of increasingly accurate models for predicting the efficacy of fertility-preserving treatments.

There are several limitations to this pilot study, primarily in its small number of samples. However, the results are promising and should be confirmed in larger investigations. A further caveat is that endometrial lavage specimens displayed a relatively low mutation frequency. In addition, only 72 oncogenes and tumor suppressor genes included in the AmpliSeq Cancer Hotspot Panel v2 were examined. As the use of office hysteroscopy advances, standardization on sampling methodology and endometrial fluid processing is expected to occur in the future. More sensitive, accurate, and timely identification of the genetic mutations in endometrial lavage specimens may hold promise for improving the clinical management of women with suspected endometrial lesions. Finally, longer follow-up is necessary to establish the predictive value of these mutations in relation to endometrial cancer risk.

## Conclusion

In conclusion, our study, albeit performed with a relatively small number of samples, indicates that mutation detection by massively parallel sequencing of endometrial lavage specimens is feasible and may allow tailoring of endometrial biopsies to the individual risk of AEH. Our findings may also pave the way toward less invasive surveillance protocols in patients with known EH. Integration of molecular signatures may allow for the development of increasingly accurate models for predicting the efficacy of fertility-preserving treatments.

## Data availability statement

The datasets presented in this study can be found in online repositories. The names of the repository/repositories and accession number(s) can be found in the article/Supplementary material.

## Ethics statement

The studies involving human participants were reviewed and approved by the Research Ethics Board at the Chang Gung Memorial Hospital (identifier: 202001329B0). The patients/participants provided their written informed consent to participate in this study.

## Author contributions

AC: study concept and design. CW, K-YW, C-JW, H-JH, C-LT, C-YL, AR, C-HL, and A-SC: data collection and interpretation. R-CW: pathological examinations. AC, CW, and R-CW: manuscript drafting and critical revision of the manuscript for important intellectual content. All authors read and approved the manuscript.

## Supplementary material

Supplementary Table 1 Summary of the 72 oncogenes and tumor suppressor genes included in the AmpliSeq Cancer Hotspot Panel v2.

Supplementary Table 2 Summary of gene mutations and allele frequencies identified in endometrial lavage samples obtained from women with atypical and non-atypical endometrial hyperplasia.

Supplementary Table 3 Summary of gene mutations and allele frequencies identified in endometrial tissue specimens obtained from women with atypical and non-atypical endometrial hyperplasia.

Supplementary Table 4 Summary of gene mutations and allele frequencies identified in endometrial tissue specimens (frequency: < 5%) obtained from women with non-atypical endometrial hyperplasia (EH19). The same mutations identified in the corresponding lavage samples had a high allele frequency.
