# Supplementary material for: Massively parallel sequencing of endometrial lavage specimens for the detection of cancer-associated mutations in atypical and non-atypical endometrial hyperplasia
Source: Front Med (Lausanne). 2022 Dec 22;9:1090788. doi: 10.3389/fmed.2022.1090788 (PMC9813484; doi:10.3389/fmed.2022.1090788)
Supplement: Supplementary Table 1 — Summary of the 72 oncogenes and tumor suppressor genes included in the AmpliSeq Cancer Hotspot Panel v2. [file Table_1.DOCX]

**Supplementary Table 1_72 genes**

| Gene ID | Official  Symbol | Also known as | Gene ID | Official  Symbol | Also known as |
| --- | --- | --- | --- | --- | --- |
| 207 | *AKT1* |  | 4916 | NTRK3 |  |
| 238 | *ALK* |  | 4921 | DDR2 |  |
| 324 | *APC* |  | 5156 | PDGFRA |  |
| 472 | *ATM* |  | 5290 | *PIK3CA* | *p110-alpha* |
| 577 | *ADGRB3* |  | 5294 | *PIK3CG* |  |
| 673 | *BRAF* |  | 5295 | *PIK3R1* | *p85-ALPHA* |
| 862 | *RUNX1T1* |  | 5296 | *PIK3R2* | *p85-BETA* |
| 1029 | *CDKN2A* | *P16INK4A* | 5314 | *PKHD1* |  |
| 1030 | *CDKN2B* | *p15INK4b* | 5604 | *MAP2K1* |  |
| 1387 | *CREBBP* | *CBP* | 5728 | *PTEN* |  |
| 1499 | *CTNNB1* |  | 5789 | *PTPRD* |  |
| 1956 | *EGFR* | *ERBB1* | 5915 | *RARB* |  |
| 2044 | *EPHA5* |  | 5925 | *RB1* |  |
| 2064 | *ERBB2* | *HER-2, NEU* | 5979 | *RET* |  |
| 2066 | *ERBB4* |  | 6016 | *RIT1* |  |
| 2260 | *FGFR1* |  | 6098 | *ROS1* |  |
| 2261 | *FGFR3* |  | 6597 | *SMARCA4* |  |
| 2263 | *FGFR2* |  | 6657 | *SOX2* |  |
| 2272 | *FHIT* |  | 6794 | *STK11* | *LKB1* |
| 2918 | *GRM8* |  | 7128 | *TNFAIP3* |  |
| 3265 | *HRAS* |  | 7157 | *TP53* | *p53* |
| 3717 | *JAK2* |  | 7248 | *TSC1* |  |
| 3791 | *KDR* | *VEGFR3* | 7307 | *U2AF1* |  |
| 3815 | *KIT* | *CD117* | 8085 | *KMT2D* |  |
| 3845 | *KRAS* |  | 8241 | *RBM10* |  |
| 4089 | *SMAD4* | *MADH4* | 8289 | *ARID1A* |  |
| 4193 | *MDM2* |  | 8314 | *BAP1* |  |
| 4233 | *MET* |  | 9817 | *KEAP1* |  |
| 4292 | *MLH1* |  | 11186 | *RASSF1* |  |
| 4609 | *MYC* |  | 23269 | *MGA* |  |
| 4763 | *NF1* |  | 25793 | *FBXO7* |  |
| 4780 | *NFE2L2* |  | 29072 | *SETD2* |  |
| 4851 | *NOTCH1* |  | 53353 | *LRP1B* |  |
| 4893 | *NRAS* |  | 55294 | *FBXW7* |  |
| 4914 | *NTRK1* |  | 94025 | *MUC16* |  |
| 4915 | *NTRK2* |  | 139285 | *AMER1* |  |

**Supplementary Table 2_Lavage (VMF>1%)**

| **Sample**  **name** | **Pathology** | **Sample** | **Gene** | **Exon** | **Chr** | **Position** | **DNA**  **Change** | **AA**  **Change** | **Mutation annotation** | **DNA reference** | **DNA variant** | **VMT** | **UMT** | **VMF** | **Total counts** |
| --- | --- | --- | --- | --- | --- | --- | --- | --- | --- | --- | --- | --- | --- | --- | --- |
| EH01L | AEH | fluid | *PTEN* | exon5 | chr10 | 89692792 | c.C276A | p.D92E | missense | C | A | 49 | 323 | 15.17% | 1320 |
| EH02L | AEH | fluid | *CTNNB1* | exon3 | chr3 | 41266097 | c.G94T | p.D32Y | missense | G | T | 31 | 1858 | 1.67% | 4636 |
| EH02L | AEH | fluid | *FGFR2* | exon3 | chr10 | 123298136 | c.A373G | p.I125V | missense | T | C | 35 | 1846 | 1.90% | 4949 |
| EH03L | AEH | fluid | *CTNNB1* | exon3 | chr3 | 41266101 | c.C98G | p.S33C | missense | C | G | 46 | 1817 | 2.53% | 3197 |
| EH03L | AEH | fluid | *CTNNB1* | exon3 | chr3 | 41266112 | c.T109G | p.S37A | missense | T | G | 93 | 1815 | 5.12% | 3232 |
| EH03L | AEH | fluid | *PIK3CA* | exon20 | chr3 | 178948100 | c.C2872A | p.Q958K | missense | C | A | 69 | 2014 | 3.43% | 4723 |
| EH04L | AEH | fluid | *AKT1* | exon3 | chr14 | 105246482 | c.G118A | p.E40K | missense | C | T | 36 | 2128 | 1.69% | 8437 |
| EH04L | AEH | fluid | *FBXW7* | exon9 | chr4 | 153247367 | c.C1081G | p.R361G | missense | G | C | 33 | 2072 | 1.59% | 8177 |
| EH04L | AEH | fluid | *KRAS* | exon2 | chr12 | 25398285 | c.G34T | p.G12C | missense | C | A | 49 | 911 | 5.38% | 2955 |
| EH04L | AEH | fluid | *STK11* | exon1 | chr19 | 1207087 | c.T175G | p.S59A | missense | T | G | 41 | 1734 | 2.36% | 7362 |
| EH05L | AEH | fluid | *AKT1* | exon3 | chr14 | 105246482 | c.G118A | p.E40K | missense | C | T | 245 | 1916 | 12.79% | 5002 |
| EH05L | AEH | fluid | *ARID1A* | exon20 | chr1 | 27107131 | c.6742_6745del | p.H2248Qfs*17 | frameshift | CACT | - | 79 | 965 | 8.19% | 2490 |
| EH05L | AEH | fluid | *KRAS* | exon2 | chr12 | 25398285 | c.G34T | p.G12C | missense | C | A | 160 | 685 | 23.36% | 1487 |
| EH05L | AEH | fluid | *PIK3CA* | exon10 | chr3 | 178936082 | c.G1624A | p.E542K | missense | G | A | 38 | 552 | 6.88% | 2019 |
| EH07L | AEH | fluid | *PIK3R1* | exon5 | chr5 | 67590433 | c.C406T | p.Q136X | stop_gained | C | T | 50 | 1861 | 2.69% | 4749 |
| EH09L | AEH | fluid | *TP53* | exon3 | chr17 | 7577520 | c.T365A | p.I122N | missense | A | T | 93 | 819 | 11.36% | 6117 |
| EH10L | AEH | fluid | *FBXW7* | exon9 | chr4 | 153247366 | c.G1082A | p.R361Q | missense | C | T | 86 | 2255 | 3.81% | 5550 |
| EH16L | NEH | fluid | *EPHA5* | exon13 | chr4 | 66217244 | c.C2308A | p.L770I | missense | G | T | 28 | 1468 | 1.91% | 5925 |
| EH16L | NEH | fluid | *FGFR2* | exon4 | chr10 | 123279677 | c.C410G | p.S137W | missense | G | C | 26 | 426 | 6.10% | 1592 |
| EH16L | NEH | fluid | *PIK3CA* | exon21 | chr3 | 178952084 | c.C3139T | p.H1047Y | missense | C | T | 26 | 771 | 3.37% | 2403 |
| EH17L | NEH | fluid | *PTEN* | exon5 | chr10 | 89692920 | c.405dupA | p.C136Mfs*43 | frameshift | - | A | 58 | 1414 | 4.10% | 4208 |
| EH18L | NEH | fluid | *CREBBP* | exon12 | chr16 | 3823820 | c.T2281G | p.F761V | missense | A | C | 69 | 856 | 8.06% | 2055 |
| EH18L | NEH | fluid | *TP53* | exon1 | chr17 | 7578401 | c.C133T | p.P45S | missense | G | A | 35 | 1302 | 2.69% | 2817 |
| EH19L | NEH | fluid | *AKT1* | exon3 | chr14 | 105246551 | c.G49A | p.E17K | missense | C | T | 451 | 1221 | 36.94% | 2606 |
| EH19L | NEH | fluid | *CTNNB1* | exon3 | chr3 | 41266125 | c.C122T | p.T41I | missense | C | T | 326 | 1898 | 17.18% | 3824 |
| EH19L | NEH | fluid | *LRP1B* | exon7 | chr2 | 141946045 | c.C958A | p.L320M | missense | G | T | 68 | 4235 | 1.61% | 12758 |
| EH19L | NEH | fluid | *PIK3R1* | exon4 | chr5 | 67589585 | c.259_267TATTCC | p.H87_Y89delinsYS | frameshift | CATGAATAT | TATTCC | 488 | 3434 | 14.21% | 8955 |

**Supplementary Table 3_FFPE tumor (VMF>5%)**

| **Sample**  **name** | **Pathology** | **Sample** | **Gene** | **Exon** | **Chr** | **Position** | **DNA**  **Change** | **AA**  **Change** | **Mutation annotation** | **DNA reference** | **DNA variant** | **VMT** | **UMT** | **VMF** | **Total counts** |
| --- | --- | --- | --- | --- | --- | --- | --- | --- | --- | --- | --- | --- | --- | --- | --- |
| EH01T | AEH | FFPE tumor | *PTEN* | exon5 | chr10 | 89692792 | c.C276A | p.D92E | missense | C | A | 263 | 1261 | 20.86% | 4481 |
| EH02T | AEH | FFPE tumor | *PTEN* | exon5 | chr10 | 89692923 | c.G407T | p.C136F | missense | G | T | 177 | 2980 | 5.94% | 5832 |
| EH02T | AEH | FFPE tumor | *PTEN* | exon7 | chr10 | 89717761 | c.786delC | p.N262Kfs*3 | frameshift | C | - | 302 | 5019 | 6.02% | 9710 |
| EH02T | AEH | FFPE tumor | *PTEN* | exon5 | chr10 | 89692934 | c.418_434A | p.L140Ifs*1 | frameshift | TTACATCGGGGCAAATT | A | 263 | 2917 | 9.02% | 5742 |
| EH02T | AEH | FFPE tumor | *PTEN* | exon5 | chr10 | 89692790 | c.G274T | p.D92Y | missense | G | T | 134 | 1389 | 9.65% | 4466 |
| EH02T | AEH | FFPE tumor | *CTNNB1* | exon3 | chr3 | 41266104 | c.G101A | p.G34E | missense | G | A | 386 | 3177 | 12.15% | 4320 |
| EH03T | AEH | FFPE tumor | *CTNNB1* | exon3 | chr3 | 41266101 | c.C98G | p.S33C | missense | C | G | 100 | 1975 | 5.06% | 2531 |
| EH03T | AEH | FFPE tumor | *CTNNB1* | exon3 | chr3 | 41266112 | c.T109G | p.S37A | missense | T | G | 199 | 1947 | 10.22% | 2504 |
| EH03T | AEH | FFPE tumor | *PIK3CA* | exon20 | chr3 | 178948100 | c.C2872A | p.Q958K | missense | C | A | 200 | 1903 | 10.51% | 3871 |
| EH04T | AEH | FFPE tumor | *ARID1A* | exon20 | chr1 | 27107131 | c.6742_6745del | p.H2248Qfs*17 | frameshift | CACT | - | 107 | 756 | 14.15% | 1419 |
| EH04T | AEH | FFPE tumor | *PIK3CA* | exon10 | chr3 | 178936082 | c.G1624A | p.E542K | missense | G | A | 82 | 531 | 15.44% | 1520 |
| EH04T | AEH | FFPE tumor | *AKT1* | exon3 | chr14 | 105246482 | c.G118A | p.E40K | missense | C | T | 132 | 554 | 23.83% | 1026 |
| EH04T | AEH | FFPE tumor | *KRAS* | exon2 | chr12 | 25398285 | c.G34T | p.G12C | missense | C | A | 184 | 607 | 30.31% | 968 |
| EH05T | AEH | FFPE tumor | *ARID1A* | exon20 | chr1 | 27107131 | c.6742_6745del | p.H2248Qfs*17 | frameshift | CACT | - | 120 | 1158 | 10.36% | 2123 |
| EH05T | AEH | FFPE tumor | *PIK3CA* | exon10 | chr3 | 178936082 | c.G1624A | p.E542K | missense | G | A | 53 | 397 | 13.35% | 1001 |
| EH05T | AEH | FFPE tumor | *AKT1* | exon3 | chr14 | 105246482 | c.G118A | p.E40K | missense | C | T | 401 | 1860 | 21.56% | 3339 |
| EH05T | AEH | FFPE tumor | *KRAS* | exon2 | chr12 | 25398285 | c.G34T | p.G12C | missense | C | A | 212 | 663 | 31.98% | 1066 |
| EH11T | AEH | FFPE tumor | *PIK3R1* | exon6 | chr5 | 67591132 | c.636_638del | p.T213del | frameshift | GAC | - | 195 | 1165 | 16.74% | 2124 |
| EH11T | AEH | FFPE tumor | *U2AF1* | exon2 | chr21 | 44524456 | c.C101T | p.S34F | missense | G | A | 173 | 997 | 17.35% | 1809 |
| EH11T | AEH | FFPE tumor | *KRAS* | exon2 | chr12 | 25398284 | c.G35T | p.G12V | missense | C | A | 142 | 763 | 18.61% | 1235 |
| EH11T | AEH | FFPE tumor | *PTEN* | exon6 | chr10 | 89712000 | c.618delC | p.F206Lfs*14 | frameshift | C | - | 462 | 1316 | 35.11% | 2072 |
| EH12T | AEH | FFPE tumor | *CTNNB1* | exon3 | chr3 | 41266103 | c.G100A | p.G34R | missense | G | A | 449 | 3301 | 13.60% | 4594 |
| EH20T | NEH | FFPE tumor | *PTEN* | exon7 | chr10 | 89717733 | c.T758G | p.I253S | missense | T | G | 280 | 3633 | 7.71% | 6525 |
| EH21T | NEH | FFPE tumor | *PIK3CA* | exon10 | chr3 | 178936093 | c.G1635T | p.E545D | missense | G | T | 251 | 1716 | 14.63% | 5708 |

**Supplementary Table 4_EH19_tumor (VMF 1%-5%)**

| **Sample**  **name** | **Pathology** | **Sample** | **Gene** | **Exon** | **Chr** | **Position** | **DNA**  **Change** | **AA**  **Change** | **Mutation annotation** | **DNA reference** | **DNA variant** | **VMT** | **UMT** | **VMF** | **Total counts** |
| --- | --- | --- | --- | --- | --- | --- | --- | --- | --- | --- | --- | --- | --- | --- | --- |
| EH19T | NEH | FFPE tumor | *AKT1* | exon3 | chr14 | 105246551 | c.G49A | p.E17K | missense | C | T | 72 | 1793 | 4.02% | 2327 |
| EH19T | NEH | FFPE tumor | *CTNNB1* | exon3 | chr3 | 41266125 | c.C122T | p.T41I | missense | C | T | 82 | 3640 | 2.25% | 4790 |
| EH19T | NEH | FFPE tumor | *PIK3R1* | exon4 | chr5 | 67589585 | c.259_267TATTCC | p.H87_Y89delinsYS | frameshift | CATGAATAT | TATTCC | 118 | 5985 | 1.97% | 11305 |
